# Evaluation of the Awareness and Anxiety Levels and Oral Hygiene Habits of University Students During the Coronavirus Disease 2019 (COVID-19) Pandemic

**DOI:** 10.7759/cureus.75696

**Published:** 2024-12-14

**Authors:** Emre Ertem, Mehtikar Gürsel

**Affiliations:** 1 Periodontology, Biruni University, Istanbul, TUR; 2 Periodontology, Bezmialem Vakıf University, Istanbul, TUR

**Keywords:** anxiety, covid-19 awareness, covid-19 pandemic, oral hygiene habits, university students

## Abstract

Aim: The aim of this study was to evaluate the awareness and anxiety levels and oral hygiene habits of university students during the coronavirus disease 2019 (COVID-19) pandemic.

Materials and methods: A total number of 560 university students were included at different education levels. Sociodemographic data regarding age, gender, current school level, family income, and smoking frequency status were obtained. Anxiety levels were evaluated through the Coronavirus Anxiety Scale and awareness levels through the Coronavirus Awareness Scale. Oral hygiene habits before and during the COVID-19 pandemic were also recorded. In addition, the participants were asked to evaluate their oral hygiene and general health status before and after the pandemic. Data were collected online using a data collection form consisting of five sections via the Google Forms (Google Inc., Mountain View, California, United States) platform. The data were statistically analyzed. p<0.05 was considered statistically significant.

Results: The statistical analysis revealed that there is a statistically significant difference in COVID-19 anxiety levels according to the current school level and family income (all with p<0.05). COVID-19 awareness levels differed significantly according to the current school level and family income (all with p<0.05). A statistically significant discrepancy was identified in the oral hygiene habits of the examined population according to gender, smoking frequency status, and family income prior to the onset of the pandemic (all with p<0.05). The examination of oral hygiene habits during the pandemic revealed statistically significant differences in accordance with the subjects' gender, smoking frequency status, current school level, and family income (all with p<0.05). During the pandemic, there was an increase in the frequency of tooth brushing, tongue brushing, toothbrush change, dental floss use, and acidic beverage consumption compared to the pre-pandemic period (all with p<0.05).

Conclusion: The present study examined the impact of the ongoing pandemic on oral hygiene habits among university students. The findings revealed a notable increase in the frequency of many oral hygiene habits during the pandemic compared to pre-pandemic levels. However, the study also observed that while anxiety levels were low, awareness levels were high. This study is the first to evaluate awareness and anxiety levels and oral hygiene habits in this context.

## Introduction

In late December 2019, certain cases presenting with some atypical pneumonia symptoms first appeared in Wuhan City, Hubei Province, China. Afterwards, several independent centers identified this mysterious pneumonia-causing agent as a novel type of coronavirus (nCoV). The World Health Organization (WHO) named the disease coronavirus disease 2019 (COVID-19) after it was detected and frequently seen in more than 110 countries and declared it a pandemic on March 11, 2020 [1]. This rapidly spreading disease triggered a cascade of economic and psychological challenges across numerous nations. Beyond the global suspension of educational activities, numerous public health interventions were implemented including restrictions on movement and the enforcement of social distancing practices. During pandemics like COVID-19, the experience of panic, anxiety, and fear can increase the risk of developing depression and anxiety-related disorders. Schools are also beneficial to sustaining students' mental health since they provide opportunities for active engagement and school routines are beneficial to the mental health of young people [2,3].

Oral hygiene aims to keep the oral cavity, teeth, and gingiva clean and healthy to prevent diseases and is beneficial to a person's quality of life as well as physical and psychological health. Personal oral hygiene measures, such as tooth brushing, have been shown to improve oral health [4]. The oral cavity is known as the "window to overall health." Since oral health is intertwined with overall health, disease in one will have an impact on both. Personal oral hygiene practices include using toothbrushes, dental floss, mouthwash, and other tools to remove plaque from tooth surfaces [5]. Microorganisms in the oral biofilm can be aspirated from the respiratory tract, resulting in the onset or progression of conditions such as pneumonia or sepsis. The pandemic appears to have a significant impact on people's daily habits and routines worldwide. Most people faced significant challenges in adapting their lives to the "new normal" [6]. In addition to some of the pandemic's negative effects, people are becoming more aware that they should practice better hygiene. The lifestyles and health habits of most people underwent profound changes as a result of the measures implemented in response to the pandemic. There have been a few studies in the literature investigating the effects of the pandemic on oral hygiene habits [7-9].

In light of this information, the aim of this study was to evaluate the oral hygiene status of university students before and during the COVID-19 pandemic and to evaluate their anxiety and awareness levels regarding the COVID-19 pandemic.

## Materials and methods

Before the data collection process in the study, ethics committee approval was received from the Non-Interventional Research Ethics Committee of Biruni University dated October 22, 2021, with decision number 2021/60-10. This study was conducted at Biruni University in Istanbul between November 2021 and May 2022, when face-to-face education started after taking precautions during the pandemic.

The study population comprised associate, undergraduate, and graduate students at Biruni University who were actively engaged during the pandemic. In a simple random sampling procedure with a known population, a sample size estimation calculation was performed. In the study, it was determined that a total of 6812 students at Biruni University met the criteria for inclusion in the study, with a minimum of 421 students required at the 95% confidence interval at α=0.05 level. However, due to potential losses, it was deemed more appropriate to take this number to around 560. Ultimately, 562 university students were included in the study.

In the study, the scale technique, which is one of the quantitative data collection methods, and the survey technique were used together.

Inclusion criteria

Active associate, undergraduate, and graduate students at Biruni University during the pandemic were included in the study.

Exclusion criteria

Students who were inactive during the pandemic period were not included in the study.

Before the survey and forms were shared with the participants, an average duration was calculated with five people, and the average duration was found to be 11.12±0.87 minutes.

It was explained to the participants who volunteered to participate in the study that the information obtained from them would not be shared with anyone and that others would not be allowed to access this information. The participants were informed before the survey started that answering the survey and scales meant that they participated in the study and their responses were collected with their consent. The names of the participants were not taken due to the principle of voluntariness and confidentiality. In our survey, each question was designed to be marked as mandatory to prevent data loss. 

In the research, a semi-structured research form prepared by the researcher by scanning the literature on the subject was used as a data collection tool, and this form was divided into five sections. These sections are listed as follows: Sociodemographic Data, Coronavirus Anxiety Scale, Coronavirus Awareness Scale, Oral Hygiene Habits Before the COVID-19 Pandemic, and Oral Hygiene Habits During the COVID-19 Pandemic.

The Sociodemographic Data was comprised of questions designed to ascertain the sociodemographic characteristics of the students, including gender, income status, current school level, and smoking status.

The Coronavirus Anxiety Scale, developed by Lee, was employed for the purpose of ascertaining the degree of anxiety associated with the ongoing COVID-19 pandemic. The scale comprises five questions, with a minimum score of 0 and a maximum score of 20. The total score obtained from all items on the scale serves to indicate the individual's level of anxiety regarding the novel coronavirus. A high score on the scale is indicative of a high level of anxiety about COVID-19 [10].

The Coronavirus Awareness Scale developed by Bilgin comprises 17 questions, presented in the format of a 5-point Likert scale. The lowest possible score is 17 points, while the highest is 85 points. A high score on the scale is indicative of a high level of awareness regarding the novel coronavirus [11].

The sections Oral Hygiene Habits Before and During the COVID-19 Pandemic consisted of questions about oral care routines such as frequency of tooth brushing and flossing, questions about dentist visits and dental treatments applied, and questions in which the participant evaluates his or her own oral hygiene status.

Statistical analysis

The data obtained online with Google Forms (Google Inc., Mountain View, California, United States) were transferred to the Excel Office (Microsoft Corporation, Redmond, Washington, United States) and analyzed with IBM SPSS Statistics for Windows, Version 22.0 (Released 2013; IBM Corp., Armonk, New York, United States). First, the reliability of the data was examined. The reliability of the Coronavirus Anxiety Scale and Coronavirus Awareness Scale was subjected to the Cronbach alpha test. 

The Mann-Whitney U test was performed to compare COVID-19 anxiety and awareness levels by gender. In order to test the hypothesis that there is a significant difference between oral hygiene habits in the period before and during the onset of COVID-19, a t-test was applied to test the relationship between oral hygiene habits in the period before the pandemic, according to gender, smoking frequency status, family income, and demographic data, and to test the relationship between oral hygiene habits in the period during the pandemic, according to gender, smoking frequency status, current school level, family income, and demographic data. The independent samples t-test compared the means of two independent groups in order to determine whether there is statistical evidence that the associated population means are significantly different. The independent samples t-test is a parametric test, while the Mann-Whitney U test is a non-parametric test that is an alternative to the independent samples t-test. This test is used to look at the difference in means between two independent groups from similar populations and to determine the difference or equality between the groups. When the test features and usage areas are examined, it is seen that it is suitable for use in the analysis.

## Results

The reliability level of the Coronavirus Anxiety Scale was 0.94, while that of the Coronavirus Awareness Scale was determined as 0.94. In this context, parametric testing techniques were used in the study. Afterwards, the distribution of the sociodemographic characteristics of the participants was examined (Table 1). Most of the participants were women. A majority of the participants are currently enrolled in the university unit as associate degree students. The average family incomes of the participants revealed that the largest group is composed of 1-2 minimum wage earner individuals. The majority of participants in the study were non-smokers.

**Table 1:** Frequency and percentage findings regarding demographic characteristics

		Frequency	Percentage (%)
Gender	Female	414	73.7
	Male	148	26.3
Current school level	Associate degree	381	67.8
	Graduate	166	29.5
	Postgraduate	9	1.6
	PhD	6	1.1
Family income	1 minimum wage	132	23.5
	<1-2 minimum wage	240	42.7
	<2-3 minimum wage	105	18.7
	<3-4 minimum wage	37	6.6
	<4 minimum wage and above	48	8.5
Smoking frequency status	None	411	73.1
	Less than 10 cigarettes per day	77	13.7
	10-20 cigarettes per day	64	11.4
	20 or more cigarettes per day	10	1.8
	Total	562	100

A significant difference was identified between the oral hygiene habits of individuals before and during the pandemic with regard to several key variables, including the frequency of tooth brushing, mouthwash, tongue brushing habit, toothbrush replacement frequency, dental floss use, consumption of acidic beverages, and evaluation of one's general health (all with p<0.05). During the pandemic, a statistically significant increase was observed in the frequency of tooth brushing, tongue brushing, flossing, and acidic beverage consumption. Participants also reported a worse evaluation of one's own general health compared to the pre-pandemic period (Table 2).

**Table 2:** Differences between oral hygiene habits before and after the COVID-19 pandemic ^#^t-test. p<0.05. The symbol ^*^ indicates that the result is statistically significant. COVID-19: coronavirus disease 2019

	Before the pandemic n (%)		During the pandemic n (%)		p^#^
Frequency of tooth brushing	Once a day or less: 199 (35.4%)	Twice a day: 363 (64.5%)*	Once a day or less: 152 (27%)	Twice a day: 410 (73%)*	0.001*
Frequency of mouthwash use	Once a day or more: 384 (68.3%)	Less than one time per day: 178 (31.7%)	Once a day or more: 361 (64.2%)	Less than one time per day: 201 (35.8%)	0.009*
Tongue brushing	Yes: 371 (66%)*	No: 191 (34%)	Yes: 407 (72.4%)*	No: 155 (27.6%)	0.000*
Toothbrush replacement frequency	Once in three months: 213 (37.9%)*	Less than once every three months: 349 (62.1%)	Once in three months: 239 (42.5%)*	Less than once every three months: 323 (57.5%)	0.000*
Frequency of dental floss use	One time per day or more: 461 (82.02%)*	Less than one time per day: 101 (17.98%)	One time per day or more: 503 (89.5%)*	Less than one time per day: 59 (12.3%)	0.001*
Frequency of interdental brush use	Less than once a day: 520 (92.5%)	One time per day or more: 42 (7.5%)	Less than one time per day: 511 (90.7%)	One time per day or more: 51 (9.1%)	0.093
Frequency of oral irrigator use	Less than one time per day: 490 (87.2%)	One time per day or more: 72 (12.8%)	Less than one time per day: 496 (88.3%)	One time per day or more: 66 (11.7%)	0.418
Consumption of sweets	None or little: 463 (82.4%)	Moderate and very much: 99 (17.6%)	None or little: 449 (79.9%)	Moderate and very much: 113 (20.1%)	0.109
Consumption of fizzy drinks	None or little: 449 (79.9%)	Moderate and very much: 113 (20.1%)*	None or little: 332 (59.1%)	Moderate and very much: 230 (40.9%)*	0.029*
Frequency of visiting the dentist	Regularly: 447 (79.5%)	Irregular: 115 (20.5%)	Regularly: 460 (81.9%)	Irregular: 102 (18.1%)	0.098
Thoughts on dental treatment	I am not afraid: 293 (52.1%)	I'm afraid: 269 (47.9%)	I am not afraid: 300 (53.4%)	I'm afraid: 262 (46.6%)	0.790
Evaluation of one's own general health	Good: 474 (84.3%)	Medium-weak: 88 (15.7%)*	Good: 410 (73%)	Medium-weak: 152 (27%)*	0.000*
Evaluation of one's own oral health	Good: 464 (82.6%)	Medium-weak: 98 (17.4%)	Good: 459 (81.7%)	Medium-weak: 103 (18.3%)	0.620

When pre-pandemic oral hygiene habits were examined, statistically significant differences were observed according to gender, smoking frequency status, and family income demographic data. It was determined that there were significant differences between genders in terms of frequency of mouthwash use, tongue brushing, frequency of interdental brush use, sweet consumption, thoughts about dental treatment, and person's evaluation of oral health from oral hygiene habits before the COVID-19 pandemic (all with p<0.05). In women, the frequency of tongue brushing, frequency of sweet consumption, fear of dental treatment, and evaluation of oral health as good scores were found to be higher, while the frequency of mouthwash use was found to be lower. It was determined that there were significant differences in the items thoughts about dental treatment and person's evaluation of oral health from oral hygiene habits according to smoking frequency status before the pandemic (all with p<0.05). The group who smoked 20 or more cigarettes was less afraid of dental treatment. As smoking increased, people evaluated their own oral hygiene as worse than those who consumed less or did not use it at all. It was determined that there was a significant difference in the frequency of dental floss from oral hygiene habits before the pandemic according to family income (all with p<0.05). As family income level increased, the frequency of dental floss use increased (Tables 3-5). Only statistically significant ones have been mentioned in the tables.

**Table 3:** Differences in oral hygiene habits before the COVID-19 pandemic by gender p<0.05. The symbol ^*^ indicates that the result is statistically significant. ^#^t-test. COVID-19: coronavirus disease 2019

Scale items	Gender		p^#^
	Women n (%)	Men n (%)	
Tongue brushing (Yes)	296 (71%)	75 (50%)	0.000*
Interdental brush usage (One time per day or more)	38 (8%)	4 (2%)	0.010*
Consumption of sweets (Moderate and very much)	84 (20%)	15 (10%)	0.000*
Fear of dental treatment (Afraid)	212 (51%)	57 (38%)	0.008*
Evaluating one's oral health (Good)	364 (87%)	100 (67%)	0.002*

**Table 4:** Differences in oral hygiene habits before the COVID-19 pandemic by smoking frequency status p<0.05. The symbol ^*^ indicates that the result is statistically significant. Only statistically significant ones are tabulated. ^¶^Compared with other groups. ^#^t-test. COVID-19: coronavirus disease 2019

Scale items	Smoking frequency status				p^#^
	No n (%)	Less than 10 cigarettes n (%)	10-20 cigarettes n (%)	20 cigarettes or above n (%)	
Fear of dental treatment (Afraid)	204 (65.5%)	41 (53.2%)	22 (34.3%)	2 (20%)^¶^	0.037*
Oral health of the person positive evaluation (Good)	355 (86.3%)	54 (70.12%)	41 (64%)	4 (40%)^¶^	0.000*

**Table 5:** Differences in oral hygiene habits before the COVID-19 pandemic by family income p<0.05. The symbol ^*^ indicates that the result is statistically significant. Only statistically significant ones are tabulated. ^†^Compared with other groups. ^#^t-test. COVID-19: coronavirus disease 2019

Scale items	Family income					p^#^
	1 minimum wage n (%)	<1-2 minimum wage n (%)	<2-3 minimum wage n (%)	<3-4 minimum wage n (%)	4 minimum wage or above n (%)	
Use of dental floss (One time per day or more)	77 (58.3%)	186 (77.4%)	83 (79%)	38 (84.4%)	42 (87.4%)^†^	0.017*

When oral hygiene habits were examined during the pandemic, differences were found according to gender, current degree, family income, and smoking. During the period of the pandemic, women were more likely to engage in twice-daily tooth brushing, tongue brushing, and the consumption of moderate to high amounts of sugar (all with p<0.05). The group with doctoral education exhibited a greater proclivity for altering their toothbrush frequency to a quarterly interval, engaging in daily flossing, utilizing mouthwash on a daily basis, appraising their general health as favorable, and consuming less frequent quantities of sugar- and carbonated beverage-based products (all with p<0.05). As the average family income level rises, so too does the frequency of flossing once a day or more and self-assessment of good health. Conversely, the frequency of moderate or high consumption of carbonated beverages and sugar tends to decline (all with p<0.05). The group who smoked 20 or more cigarettes per day exhibited a lower frequency of brushing their teeth twice a day, a lower frequency of rating their general health as good, a lower frequency of rating their oral health as good, and a higher frequency of fear of dental treatment compared other groups (Tables 6-8) (all with p<0.05). Only statistically significant ones have been mentioned.

**Table 6:** Differences in oral hygiene habits during the COVID-19 pandemic by gender p<0.05. The symbol ^*^ indicates that the result is statistically significant. Only statistically significant ones are tabulated. ^#^t-test. COVID-19: coronavirus disease 2019

Scale items	Gender		p^#^
	Women n (%)	Men n (%)	
Brushing teeth (Twice a day)	326 (78%)	84 (56%)	0.000*
Tongue brushing (Yes)	330 (79%)	87 (58%)	0.028*
Consumption of sweets (Moderate and very much)	342 (82%)	107 (72%)	0.007*

**Table 7:** Differences in oral hygiene habits during the COVID-19 pandemic by smoking frequency status and current school level p<0.05. The symbol ^* ^indicates that the result is statistically significant. Only statistically significant ones are tabulated.^ ¶^Compared to other income groups. ^†^Compared to other groups. ^§^Compared to other groups.^ #^t-test. COVID-19: coronavirus disease 2019

Smoking frequency status								
Scale items	No n (%)		Less than 10 cigarettes n (%)		10-20 cigarettes n (%)	20 cigarettes or above n (%)		p^#^
Brushing teeth (Twice a day)	314 (76.3%)		54 (62%)		38 (59.3%)	4 (40%)^§^		0.011*
Fear of dental treatment (Afraid)	185 (45%)		29 (37.6%)		40 (62.4%)	8 (80%)^§^		0.003*
A positive evaluation of one's general health	320 (77.8%)		42 (54.5%)		43 (67.1%)	5 (50%)^§^		0.000*
It positively affects one's oral health evaluation	359 (87.3%)		54 (70.1%)		40 (62.4%)	6 (60%)^§^		0.038*
Current school level								
Scale items	Associate degree n (%)	License n (%)		Postgraduate n (%)			PhD n (%)	p^#^
Changing toothbrushes (Once in three months)	194 (50%)	117 (70%)		7 (77%)			5 (83%)^†^	0.001*
Use of dental floss (One time per day or more)	331 (85%)	148 (87%)		8 (88%)			6 (100%)^†^	0.006*
Use of mouthwash (Once a day or more)	40 (10%)	19 (13%)		3 (33%)			4 (66%)^†^	0.004*
Consumption of sweets (Moderate and very much)	80 (20%)	30 (18%)		1 (11%)			0 (0%)^ †^	0.036*
Consumption of fizzy drinks (Moderate and very much)	173 (45%)	73 (43%)		2 (22%)			1 (16%)^†^	0.000*
Evaluating the person's general health (Good)	266 (68%)	129 (77%)		8 (88%)			6 (100%)^†^	0.026*

**Table 8:** Differences in oral hygiene habits during the COVID-19 pandemic by family income p<0.05. The symbol ^*^ indicates that the result is statistically significant. Only statistically significant ones are tabulated. ^¶^Compared to other income groups. ^#^t-test. COVID-19: coronavirus disease 2019

Scale items	Family income					p^#^
	1 minimum wage n (%)	<1-2 minimum wage n (%)	<2-3 minimum wage n (%)	<3-4 minimum wage n (%)	4 minimum wage or above n (%)	
Use of dental floss (One time per day or more)	111 (84%)	216 (89.9%)	95 (90.4%)	34 (91.8%)	46 (93.8%)^¶^	0.001*
Consumption of sweets (Moderate and very much)	115 (87.1%)	196 (81.6%)	83 (79%)	26 (74.2%)	29 (60.4%)^¶^	0.003*
Consumption of fizzy drinks (Moderate and very much)	104 (74.9%)	135 (56.2%)	43 (45.2%)	16 (43.2%)	15 (31.2%)^¶^	0.010*
A positive evaluation of one's general health	70 (71.2%)	187 (77.9%)	82 (78%)	29 (82.8%)	42 (87.4%)^¶^	0.028*

The mean anxiety score for the participants in response to COVID-19 was 1.848±3.307, with a minimum score of 0 and a maximum score of 20. The mean COVID-19 awareness score of the participants was 56.674±12.139, with a minimum score of 20 and a maximum score of 85.

When the Coronavirus Awareness Scale was examined, it was observed that the current school level and average family income created significant differences. The PhD group demonstrated a higher level of awareness regarding the novel COVID-19 awareness score than the associate degree group. The group receiving 3-4 minimum wage had higher COVID-19 awareness scores than the other groups. When the Coronavirus Anxiety Scale was examined, it was observed that current school level and average family income created significant differences. The data indicated that the anxiety scores of the doctoral education group were higher than those of the associate and postgraduate groups. The group receiving 3-4 minimum wage had higher anxiety awareness scores than the other groups (Table 9) (all with p<0.05). Only statistically significant ones have been mentioned.

**Table 9:** Differences in the Coronavirus Awareness and Anxiety Scales according to current school level and median family income ^§^Compared to associate degree.^ ¶^Compared to associate and postgraduate degrees. ^†^Compared to 1, 1-2, and 2-3 minimum wage; p<0.05. The symbol ^*^ indicates that the result is statistically significant. Only statistically significant ones are tabulated. ^#^Mann-Whitney U test.

Current school level				
Level	Awareness score min-max (median)	p^#^	Anxiety score min-max (median)	p^#^
Associate degree	20-85 (56)	0.028*	0-20 (0)	0.023*
Graduate	28-85 (58)		0-20 (0)	
Postgraduate	40-71 (59)		0-7 (1)	
Doctorate^§^	42-85 (64)		0-5 (1.5)	
Family income				
Level	Awareness score min-max (median)	p^#^	Anxiety score min-max (median)	p^#^
1 minimum wage	28-82 (58)	0.039*	0-20 (0)	0.039*
<1-2 minimum wage	26-85 (56)		0-20 (0)	
<2-3 minimum wage	20-85 (58)		0-20 (0)	
<3-4 minimum wage^†^	38-81 (60)		0-18 (1)	

## Discussion

In our study, the effect of the COVID-19 pandemic on the oral hygiene habits of university students was examined, and it was shown that there was a significant increase in the frequency of many oral hygiene habits during the pandemic compared to before. However, it was observed that COVID-19 anxiety levels were very low, while awareness levels were high. Our study is the first study evaluating anxiety and awareness levels and oral hygiene habits.

The present study was designed to evaluate oral care practices and anxiety and awareness levels of university students during the COVID-19 pandemic. Due to the selection of a group consisting of students studying at Biruni University, our data may differ from the general population due to the level of education of the participants. However, this group provides important information about how young individuals perceive the COVID-19 pandemic and its effects on their oral care habits.

In our study, a comparison of oral care habits in the pre-pandemic period with those during the pandemic revealed a statistically significant difference. The present study revealed that oral care habits were performed with greater frequency during the period of the pandemic. It is hypothesized that this is due to the fact that greater importance was attributed to oral care, in addition to general body hygiene and care, during the period of the pandemic. In a cross-sectional study, the frequency of tooth brushing of patients applying to the oral and dental health center before and during the pandemic was determined. The study was conducted with a total of 203 participants. The findings revealed that the frequency of tooth brushing at night increased during the pandemic [12]. In a related study, the oral hygiene of individuals during the pandemic was examined, and it was determined that attention to oral hygiene and the frequency of tooth brushing increased during the pandemic compared to before [12]. 

Coulthard et al. [13] attribute the observed changes in eating habits during the pandemic to anxiety and psychological factors. While some individuals adopted a healthier and more balanced diet, others experienced irregular eating patterns due to stress and anxiety. These findings align with the results of our study [13].

The findings of our study indicated that the group of participants who smoked 20 or more cigarettes per day demonstrated a lower frequency of oral hygiene procedures. The study conducted by Santos et al. [14] similarly observed a reduced frequency of tooth brushing and flossing habits among a group of 20 or more cigarette smokers, and the data from both studies align with one another [14].

This study revealed a positive correlation between oral care habits and income levels. Oberoi et al. [15] also found a significant and positive relationship between income level and oral hygiene. The results of our study align with those of Oberoi et al. It is evident that changing oral care products more frequently and using better-quality care products will vary depending on income level [15].

Our study indicated that individuals with PhD-level education engage in oral hygiene procedures with greater frequency. In their study, Paulander et al. [16] investigated the relationship between the educational levels of individuals in different age groups and their oral health. Their findings revealed that individuals with higher educational levels demonstrated a greater awareness of and commitment to oral hygiene, which was associated with superior oral health outcomes [16]. In a parallel study, the relationship between the oral care habits of patients aged 31-40 years and their education level was examined. The findings indicated that the level of education had a direct impact on the level of attention paid to oral hygiene and oral health [17].

During the course of the pandemic, there has been a notable increase in general health awareness among individuals, accompanied by a concomitant rise in the importance attached to hygiene and care habits. In particular, information regarding the transmission routes of COVID-19 has resulted in a notable increase in the adoption of careful hygiene habits. This may have resulted in an increase in the number of oral care water applications. In the study by Singh et al. [18], it was stated that adherence to hygiene measures during the period of the pandemic led to an increase in general health concerns and awareness of protection from infectious diseases. It is hypothesized that this heightened awareness may have resulted in a greater frequency of use of products that were not previously used regularly, such as mouthwash and dental floss [18].

Our study revealed that women were more likely to engage in oral hygiene practices than men. Sakki et al. [19] investigated the tooth brushing habits of a sample of 780 adult individuals in Finland. Their findings corroborate those of the present study, indicating that women were more likely to brush their teeth on a regular basis. This discrepancy may be attributed to the fact that women tend to place a higher value on oral care, given that their social and aesthetic concerns are more pronounced than those of men [19].

The findings of our study indicated that the level of awareness of COVID-19 was influenced by the current school level. Participants who had received doctoral education demonstrated a higher level of awareness than those who had not. This observation can be attributed to the general increase in awareness associated with higher levels of education. The findings of our study indicate that the group with the highest income level exhibited the highest levels of awareness. This is consistent with the results of the study conducted by Chandra et al., which demonstrated that an increase in income level is associated with enhanced health awareness. Our results provide further support for this conclusion [20].

The findings of our study indicated that the level of anxiety of COVID-19 was influenced by the current school level. In our study, individuals with doctoral-level education exhibited elevated anxiety scores. In a study by Joannès et al. [21], anxiety scores of individuals with graduate education were higher in women. The reason why individuals with doctoral education in our study had higher anxiety scores may be attributed to the challenges inherent in the educational process [21].

The findings of this study must be considered in light of its inherent limitations. Among these limitations is the fact that all data were collected from students at Biruni University, with the majority of participants being enrolled in associate degree programs. It is therefore possible that the results may differ in studies conducted with larger samples.

## Conclusions

Given all these findings, it is possible to conclude that the COVID-19 pandemic has caused some changes in people's lives, as well as an impact on their general health, anxiety levels, and oral care. This study examined the impact of the ongoing pandemic on oral hygiene habits among university students. The findings revealed a significant increase in the frequency of many oral hygiene habits during the pandemic compared to before the pandemic. However, the study also observed low levels of anxiety and high levels of awareness, which is the first study to evaluate anxiety and awareness levels and oral hygiene habits in this context.
